# Comparison of the Incidence of Sore Throat After Rapid Sequence Intubation With Succinylcholine and Cisatracurium

**DOI:** 10.5812/aapm.20030

**Published:** 2014-08-13

**Authors:** Farhad Solatpour, Houman Teymourian, Seyed Amir Mohajerani, Fatemeh Hoseinzadegan Shirazi, Saran Lotfollah Zadeh, Maryam Baikpour, Razie Amraei

**Affiliations:** 1 Department of Anesthesiology, Shohada-e-Tajrish Hospital, Shahid Beheshti University of Medical Sciences, Tehran, Iran

**Keywords:** Cisatracurium, Intratracheal Intubation, Pharyngitis, Succinylcholine

## Abstract

**Background:**

Postoperative sore throat is a common complication of endotracheal intubation and can lead to dissatisfaction after surgery. Airway management has the strongest influence on the incidence of sore throat and improving endotracheal intubating conditions can reduce this complaint. Type of induction agent used during anesthesia can contribute to variances in the degree of post-operative sore throat.

**Objectives:**

We aimed to compare the incidence of postoperative sore throat after rapid sequence induction with Succinylcholine and high dose Cisatracurium.

**Patients and Methods:**

The study was carried out on patients admitted to Shohada-e-Tajrish hospital for emergent abdominal surgery. Of the 80 patients who were enrolled in the study, 40 were randomly assigned to receive Succinylcholine while the remaining patients received Cistracurium during induction. Sore throat, muscle ache, hoarseness, dry throat and pain were assessed in each patient at baseline in recovery and at 2, 4, 12 and 24 hours post-operation.

**Results:**

Number of patients who developed sore throat was significantly higher in the Succinylcholine group (75%) compared to Cisatracurium group (27.5%) at the time of entrance to the recovery room (P = 0.001). These numbers decreased at 2 hours post–operation (42% versus 17.5%) but the difference was still statistically significant (P < 0.05). At 12 (P = 0.062) and 24 (P = 0.14) hours post operation, the difference was no longer significant.

**Conclusions:**

Use of high dose Cisatracurium for induction during rapid sequence intubation carries a lower chance of developing sore throat compared to Succinylcholine. Studies comparing other adverse effects of these two agents are required to guide physician's choice of induction agent.

## 1. Background

Sore throat is a common postoperative complaint occurring most often as a complication of endotracheal intubation with an incidence ranging from 14 to 50% (1, 2). The symptom usually lasts for about 12-24 hours post-operation. Among the causative factors airway management has the strongest influence on the incidence of sore throat (3); therefore improving endotracheal intubating conditions can reduce post operative sore throat. Several methods have been proposed to minimize this side effect: application of smaller size ETT (4), use of cuffed ETT (5), minimizing ETT cuff pressure (6), application of Lidocain gel on ETT (7), spraying the endotracheal tube cuff with benzydamine hydrochloride (8), adminisration of beclomethasone spray (9), postponing intubation until achieving complete muscle relaxation (10), mild suctioning of mouth and pharynx before extubation, and withholding extubation until the cuff is completely empty.

Factors such as Succinylcholine administration also contribute to the occurrence of postoperative sore throat (11), even though use of muscle relaxants during anesthesia has been shown to reduce post-intubation symptoms (12). It has been proposed that the type of induction agent used during anesthesia can affect the incidence of post operative sore throat. Currently, succinylcholine is the muscle relaxant of choice for rapid sequence intubation in emergency situations, but Cisatracurium administered at high doses can also be used for induction and studies comparing these two agents show that some of the adverse effects of Succinylcholine such as hyperkalemia and malignant hyperthermia are rarely seen with Cisatracurium (13). Most of the currently available literature on postoperative sore throat revolve around Succinylcholine's effect on this complication, and so far no study has been carried out to compare the incidence of sore throat after with these two agents.

## 2. Objectives

We aimed to compare the incidence of postoperative sore throat after rapid sequence induction with Succinylcholine and high dose Cisatracurium.

## 3. Patients and Methods

**Figure 1.**
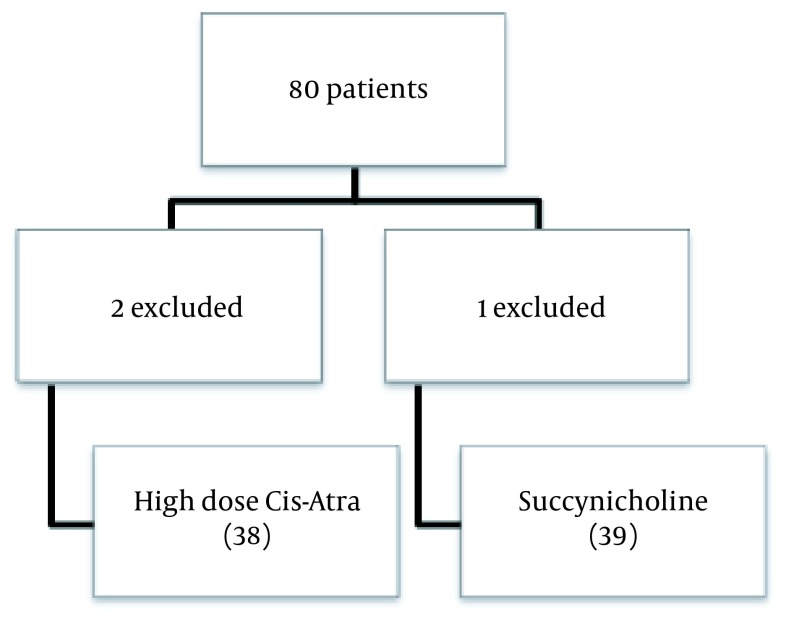
Consort Flow Diagram of the Trial

We conducted a randomized double blind clinical trial on patients admitted to Shohada-e-Tajrish hospital for emergent abdominal surgery. Patients with ASA PS (American Society of Anesthesiologists Physical Status) class I-II and aged 20 to 60 years old undergoing emergent abdominal surgery were included in the study. Exclusion criteria consisted of history of previous sore throat, cigarette smoking, history of addiction, history of heart or lung disease, previous neck surgery, mallampati score 3 or 4, difficult intubation and decline in oxygen saturation to less than 95% during induction due to difficult intubation. Consort flowchart of the study is depicted in Figure 1.

80 patients were enrolled in the study and were randomly assigned to receive either Succinylcholine or Cisatracurium. Sore throat, muscle ache, hoarseness, dry throat and pain were assessed in each patient at baseline in recovery and at 2, 4, 12, and 24 hours post-operation. Presence of sore throat, muscle ache, hoarseness and dry throat were evaluated using a simple yes/no question. Post-operative sore throat was also scaled according to visual analogue score (VAS). Patients were observed for the development of fasciculation.

General anesthesia was administered with patients in supine position. For premedication, 2 µg/kg Fentanyl and 0.02 mg/kg Midazolam was administered. For induction, 5 mg/kg Thiopental was administered. After 60-90 seconds when TOF (Train of Four) = 0 patients received either 1.5 mg/kg Succinylcholine or 0.4 mg/kg Cisatracurium, and were intubated. All intubations were performed by a single anesthesiologist. Endotracheal tubes used in the study were made in Germany and their size was selected by the anesthesiologist after direct laryngoscopy. If intubation was difficult or was attempted more than once, patient was excluded from the study. Endotracheal tube cuff was inflated until no exhalation sounds or leak were heard. Besides, inflated cuffs were measured using a standard gage at 20-30 cm H_2_O. Anesthesia was maintained using Isofluorane and Atracurium. 2 μg/kg Fentayl was repeated 1 hour after the start of the surgery. No additional analgesics were administered during the operation and patients who required this intervention were excluded from the study. Extubation was performed post-operatively using the same method in both groups. When TOF > 0.7 and the patient were fully awake, neuromuscular block was reversed using Neostigmin 0.05 mg/kg and Atropin 0.02 mg/kg, and patient was extubated. While in recovery, patients received oxygen at a rate of 5 liters/minute. If VAS ≥ 4 at any time, then analgesics were administered.

The study was reviewed and approved by the university's Ethics Committee. Comprehensive information about the trial was provided to the patients both orally and in written form. Patient enrollment was voluntary. Informed consent was obtained from all patients prior to inclusion in the study.

Sample size was calculated based on a confidence of 5% and desired power of 80% and desired ratio of 30%. In our study, based on reduction of 3 scores of VAS in postoperative sore throat the difference between Cisatracurium and Succinylcholine was calculated about 30%. Sample size calculation used the normal approximation to the Binomial distribution.

Data analysis was conducted using SPSS v.18. The parametric variables were presented as mean ± SD and were analyzed by student t-test or ANOVA and Pearson correlation test as appropriate. Statistical analysis was performed using Chi-Square or Mann-Whitney U-test and Spearman correlation coefficients for non-parametric samples. P < 0.05 was considered as statistically significant. Sample size was estimated using sample size calculator software with 95% confidence interval and P < 0.05.

## 4. Results

40 patients received Succinylcholine and 40 received Cisatracurium. There were no significant differences in the distribution of age, sex, and BMI between the two arms (Table 1). Duration of surgery was not significantly different as well (P value = 0.32).

**Table 1.** Demographic Characteristics of Patients in the Two Arms ^a^

	Succinylcholine	Cisatracurium	P Value
**Age, y**	35.5 ± 12.4	36.2 ± 14.1	0.22
**Gender**			> 0.05
Male	31	28	
Female	19	22	
**Weight, kg**	65.6 ± 21.2	71.8 ± 25.5	0.14
**BMI, kg/m^2^**	22.6 ± 3.5	23.5 ± 4.3	0.65
**Duration of surgery, min**	185 ± 45.3	192 ± 51.3	0.32

^a^ Data are presented as mean ± SD.

The incidence of sore throat and its variants (sore throat, dry throat, and hoarseness) was significantly higher in the Succinylcholine group (75%) compared to Cisatracurium group (27.5%) at baseline in the recovery room (P = 0.001).

**Figure 2.**
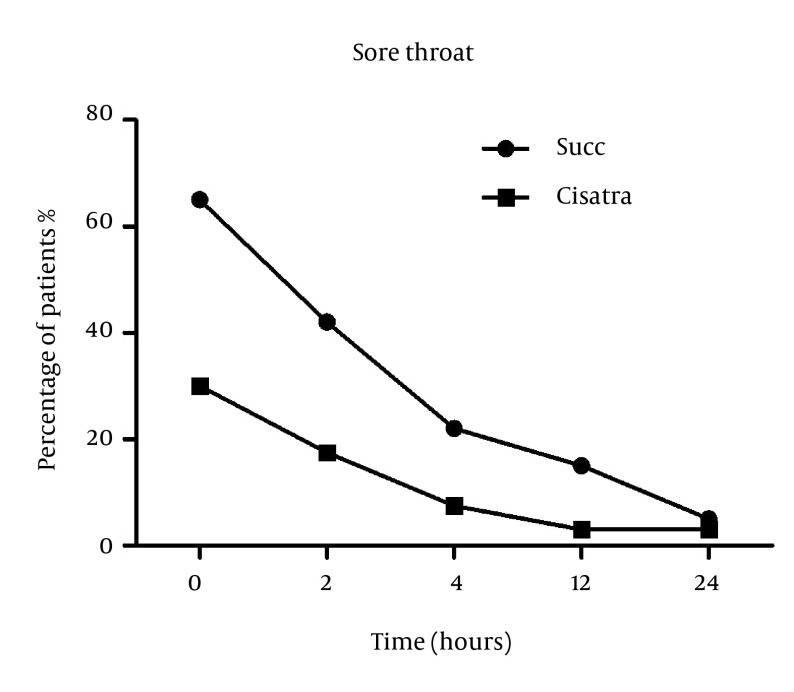
Sore Throat Incidence After Induction With Succinylcholine or Cisatracurium at Baseline, 2, 4, 12 and 24 Hours Post Operation.

Number of patients who developed sore throat was significantly higher in the Succinylcholine group (51%) compared to Cisatracurium group (25%) at the time of entrance to the recovery room (P = 0.001). These numbers decreased at 2 hours post–operation (42% in the Succinylcholine group and 17.5% in the Cisatracurium group) but the difference was still statistically significant (P = 0.027). At 12 and 24 hours post operation, the difference was no longer significant (Figure 2).

**Figure 3.**
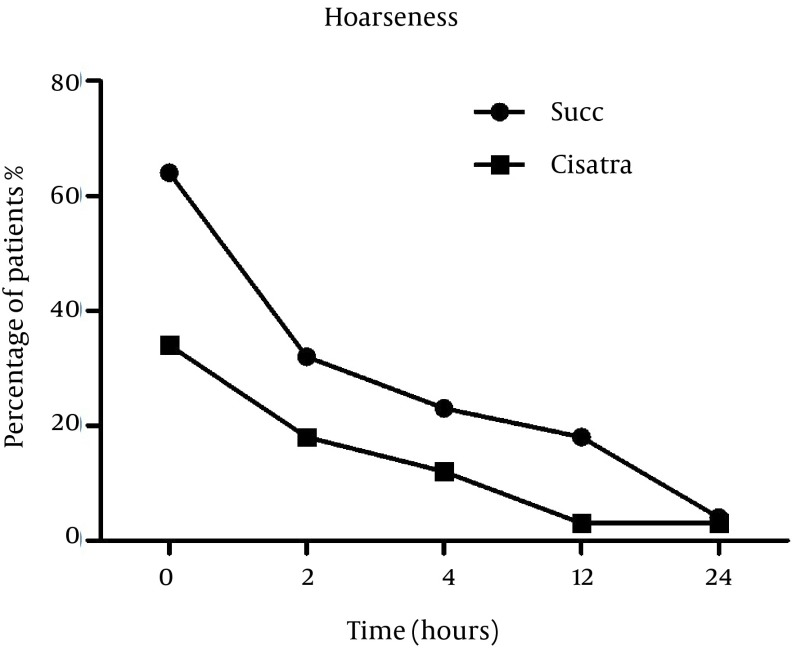
Hoarseness Incidence After Induction With Succinylcholine or Cisatracurium at Baseline, 2, 4, 12 and 24 Hours Post Operation.

Number of patients who developed hoarseness was significantly higher in the Succinylcholine group compared to Cisatracurium group at the time of entrance to the recovery room (37.5% versus 17.5% with P = 0.015) and 2 hours post–operation (15% versus 12.5% with P = 0.046) and the difference was statistically significant. At 12 and 24 hours post operation, the difference was no longer significant (P = 0.35 and P = 0.19 respectively) (Figure 3).

**Figure 4.**
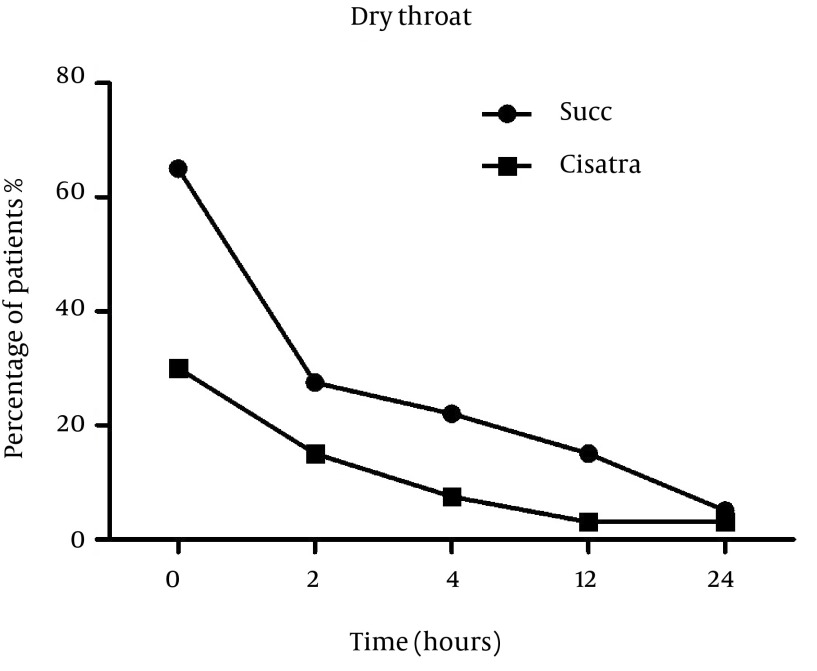
Dry Throat Incidence After Induction With Succinylcholine or Cisatracurium at Baseline, 2, 4, 12 and 24 Hours Post Operation.

Number of patients who experienced dryness of the throat was significantly higher in the Succinylcholine group (57.5%) compared to Cisatracurium group (17.5%) at baseline (57.5% compared to 17.5%) and at 2 hour post–operation (22% compared to 12.5% with P = 0.041). The difference was no longer significant at 12 (P = 0.22) and 24 (P = 0.063) hours post-operation (Figure 4).

**Figure 5.**
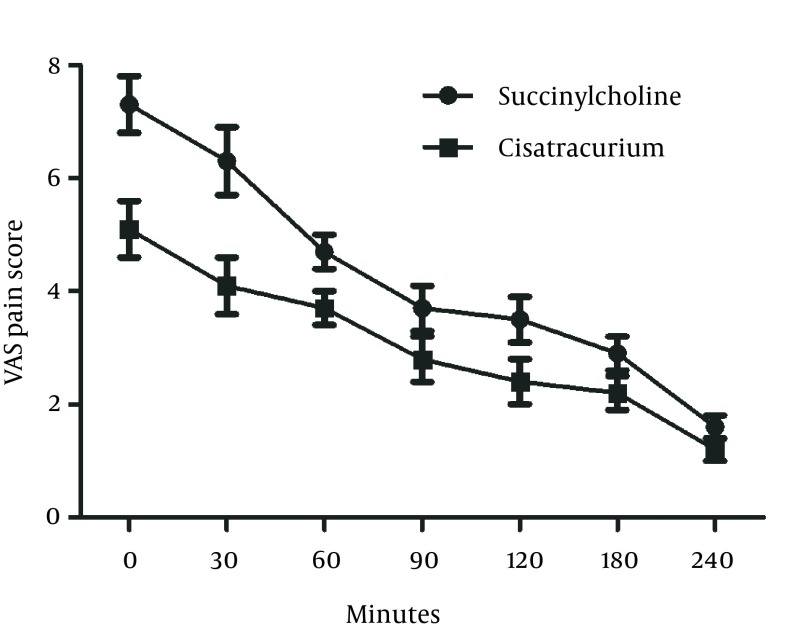
Percentage of Patients Who Experienced Pain With Visual Analogue Scale (VAS) > 3 After Induction With Succinylcholine or Cisatracurium at Baseline, 2, 4, 12 and 24 Hours Post Operation.

Number of patients with VAS > 3 was significantly higher in the Succinylcholine group (67.5%) compared to Cisatracurium group (27.5%) at the time of entrance to the recovery room. These numbers decreased at 2 hours post–operation (35.5% in the succinylcholine group and 12.5% in the Cisatracurium group) but the difference was still statistically significant (P = 0.039). At 12 (P = 0.077) and 24 (P = 0.14) hours post operation, the difference was no longer significant (Figure 5). 97.5% of the patients who had received Succinylcholine developed fasciculation, while none of the patients in the Cisatraciruim arm showed similar problem (P < 0.001).

Muscle ache and fasciculation was experienced in 85% of the patients in Succinylcholine group and 27.5% of the Cisatracurium group (P = 0.002). In order to determine the frequency of sore throat in relation to muscle ache, both arms were further divided into those who had developed muscle ache and those who didn't, and the incidence of sore throat was calculated in each group. In the Succinylcholine arm, 29.3% of patients who developed muscle ache and 28.2% of those who didn’t experienced accompanying sore throat; while in the Cisatracurium arm, these numbers were 18.5% and 14.5%, respectively (Figure 5). Number of patients with sore throat was significantly lower in the Cisatracurium arm, whether they had (P = 0.025) or had not (P = 0.01) accompanying muscle.

## 5. Discussion

As mentioned earlier, postoperative sore throat is a common complication of endotracheal intubation and can lead to dissatisfaction after surgery. Type of induction agent used for airway management has a considerable influence on the incidence of sore throat. Succinylcholine is the most commonly used type of muscle relaxant. Not many studies have evaluated the effect of Succinylcholine on postoperative sore throat, and contradictory data exists among them. A study by Capan et al. (11) published in 1983 on 83 patients undergoing dilation and curettage examined the incidence of post operative sore throat (6-10 hours post operation) after induction with different doses of Succinylcholine while avoiding perioperative intubation. According to their results, sore throat was experienced in 0% of patients who had not received Succinylcholine, 9% of patients who had received 1 mg/kg Succinylcholine and 10% of those who had received the medication at a dose of 1.5 mg/kg. The authors concluded that intravenous administration of Succinylcholine may cause postoperative sore throat (11). However, another study by Joorgensen et al. conducted on 60 patients in 1987 did not show an increase in the incidence or severity of sore throat 20 to 30 hours post operation (14). The difference can be attributed to the fact that sore throat was evaluated at different times after surgery. Our results show that the difference between the incidence of sore throat caused by Succinylcholine and Cisatracurium reduced as time went by, reaching an insignificant level 24 hours post operation. As previously stated, no study has been carried out to compare the incidence of sore throat after induction with Succinylcholine and Cisatracurium. Our results show that the number of patients who developed sore throat was significantly higher in the Succinylcholine group (75%) compared to Cisatracurium group (27.5%) at the time of entrance to the recovery room and 2 hours post–operation (42% in the Succinylcholine group and 17.5% in the Cisatracurium group) (P = 0.027).

Fasiculation has been proposed as the main cause of post-induction sore throat. Interestingly, our study showed that patients with or without fasciculation had almost equal chances of developing sore throat and the incidence varied only based on the type of muscle relaxant used: In the Succinylcholine arm 29.3% of patients who developed muscle ache and 28.2% of those who didn't, experienced accompanying sore throat; while in the Cisatracurium arm, these numbers were 18.5% and 14.5%, respectively.

We demonstrated that high dose Cisatracurium can provide a muscle relaxant effect without increasing rates of post-operative sore throat and its use during rapid sequence intubation carries a lower chance of developing sore throat compared to Succinylcholine. Studies comparing other adverse effects of these two agents are required to decide which one is generally a more suitable option for induction and to guide physicians' choices of induction agent.
